# Thioredoxin peroxidase secreted by *Echinococcus granulosus* (*sensu stricto*) promotes the alternative activation of macrophages *via* PI3K/AKT/mTOR pathway

**DOI:** 10.1186/s13071-019-3786-z

**Published:** 2019-11-14

**Authors:** Hui Wang, Chuan-Shan Zhang, Bin-Bin Fang, Zhi-De Li, Liang Li, Xiao-Juan Bi, Wen-Ding Li, Ning Zhang, Ren-Yong Lin, Hao Wen

**Affiliations:** 1 State Key Laboratory of Pathogenesis, Prevention and Treatment of High Incidence Diseases in Central Asia, Clinical Medicine Institute, The First Affiliated Hospital of Xinjiang Medical University, Ürümqi, 830054 Xinjiang People’s Republic of China; 2 Branch of The First Affiliated Hospital of Xinjiang Medical University, Changji, 831100 Xinjiang People’s Republic of China; 3 Basic Medical College, Xinjiang Medical University, Ürümqi, 830054 Xinjiang China; 4 Xinjiang Key Laboratory of Echinococcosis, The First Affiliated Hospital of Xinjiang Medical University, Ürümqi, 830054 Xinjiang China

**Keywords:** *Echinococcus granulosus* (*sensu lato*), Thioredoxin peroxidase, Excretory-secretory products, Peritoneal macrophages, Alternative activation, PI3K/AKT/mTOR pathway

## Abstract

**Background:**

Larvae of *Echinococcus granulosus* (*sensu lato*) dwell in host organs for a long time but elicit only a mild inflammatory response, which indicates that the resolution of host inflammation is necessary for parasite survival. The recruitment of alternatively activated macrophages (AAMs) has been observed in a variety of helminth infections, and emerging evidence indicates that AAMs are critical for the resolution of inflammation. However, whether AAMs can be induced by *E. granulosus* (*s.l.*) infection or thioredoxin peroxidase (TPx), one of the important molecules secreted by the parasite, remains unclear.

**Methods:**

The activation status of peritoneal macrophages (PMs) derived from mice infected with *E. granulosus* (*sensu stricto*) was analyzed by evaluating the expression of phenotypic markers. PMs were then treated *in vivo* and *in vitro* with recombinant EgTPx (rEgTPx) and its variant (rvEgTPx) in combination with parasite excretory-secretory (ES) products, and the resulting activation of the PMs was evaluated by flow cytometry and real-time PCR. The phosphorylation levels of various molecules in the PI3K/AKT/mTOR pathway after parasite infection and antigen stimulation were also detected.

**Results:**

The expression of AAM-related genes in PMs was preferentially induced after *E. granulosus* (*s.s.*) infection, and phenotypic differences in cell morphology were detected between PMs isolated from *E. granulosus* (*s.s.*)*-*infected mice and control mice. The administration of parasite ES products or rEgTPx induced the recruitment of AAMs to the peritoneum and a notable skewing of the ratio of PM subsets, and these effects are consistent with those obtained after *E. granulosus* (*s.s.*) infection. ES products or rEgTPx also induced PMs toward an AAM phenotype *in vitro*. Interestingly, this immunomodulatory property of rEgTPx was dependent on its antioxidant activity. In addition, the PI3K/AKT/mTOR pathway was activated after parasite infection and antigen stimulation, and the activation of this pathway was suppressed by pre-treatment with an AKT/mTOR inhibitor.

**Conclusions:**

This study demonstrates that *E. granulosus* (*s.s.*) infection and ES products, including EgTPx, can induce PM recruitment and alternative activation, at least in part, *via* the PI3K/AKT/mTOR pathway. These results suggest that EgTPx-induced AAMs might play a key role in the resolution of inflammation and thereby favour the establishment of hydatid cysts in the host.

## Background

Cystic echinococcosis (CE), which is caused by *Echinococcus granulosus* (*sensu lato*) at the larval stage, is regarded as a severe chronic helminthic disease with a worldwide distribution [1]. Larvae of *E. granulosus* (*s.l*.) mainly dwell in the liver and lungs of the intermediate host, where they develop into a unilocular, fluid-filled cyst containing the larval worms or protoscoleces (PSCs). The hydatid cyst can reach several centimeters in diameter and is characterized by mild local inflammation [2, 3]. After the hydatid cyst ruptures, the spillage of PSCs into the peritoneal cavity (PerC) generates new cysts that usually cause a secondary CE infection, which is also a life-threatening form of human CE [4]. Recent studies have shown that the intraperitoneal injection of PSCs into mice induces a significant cellular inflammatory response at the early stage that involves macrophage, eosinophil, neutrophil and lymphocyte infiltration [5, 6], whereas at the cyst establishment stage, the parasite induces inflammatory cell infiltration but generally does not elicit a severe inflammatory response [2, 7]. This phenomenon indicates that *E. granulosus* (*s.l*.) has the ability to skew the peritoneal immune response away from a proinflammatory response and toward an anti-inflammatory response to avoid clearance. However, the mechanism through which the parasite modulates the host inflammatory response to favor its establishment in the host is unclear.

Macrophages play a bridge role between innate and adaptive immunity and are thus critical mediators of many chronic inflammatory diseases [8]. Peritoneal macrophages (PMs), which are one of the best-studied macrophage populations, play important roles in the control of infections and inflammatory pathologies [9], and two PM subsets in the mouse PerC were recently classified: large peritoneal macrophages (LPMs) and small peritoneal macrophages (SPMs) [10]. Studies of the functional profiles of these PMs have shown that LPMs appear to play a role in the maintenance of PerC physiological conditions as alternatively activated macrophages (AAMs), whereas SPMs present a pro-inflammatory functional profile during inflammatory initiation and control infections as classically activated macrophages (CAMs) [11]. CAMs are characterized by high expression of inducible nitric oxide synthase (iNOS) and TNF-α and exhibit microbicidal properties. In contrast, AAMs, which are characterized by high expression of mannose receptor (also known as CD206), arginase-1 (Arg-1), Ym1, and Fizz1, generally exhibit anti-inflammatory properties and thus have the ability to suppress Th1-driven inflammatory pathology during helminth infections [8, 12, 13]. In addition, AAMs are critically involved in favoring susceptibility during helminth infection because the early removal of these cells leads to *Taenia crassiceps* cysticercosis clearance *in vivo* [14]. Many studies have reported that AAMs are highly activated and recruited during infection with a range of different helminths, such as *Heligmosomoides polygyrus* [15], *Trichinella spiralis* [16], *Fasciola hepatica* [17] and *Schistosoma mansoni* [18, 19]. It has been reported that *E. granulosus* (*s.l*.) infection can effectively inhibit inflammation in a murine colitis model by reducing TNF-α production and iNOS induction by CAMs [20]. However, whether and how AAMs are induced after *E. granulosus* (*s.l*.) infection remain to be clarified.

It has been demonstrated that parasite excretory-secretory (ES) products or some released parasite surface molecules can directly modulate host immune cells to promote parasite survival [21–23]. ES products derived from *E. granulosus* (*s.l*.) are well known to regulate T cell responses, dendritic cell maturation and B cell subset differentiation [24–29], but their regulation of macrophage activation is not well understood. Recent studies have shown that *E. granulosus* (*s.l*.) laminated layer (LL) extracts can induce arginase expression, a hallmark of AAMs, to counteract NO production by CAMs in mouse PMs *in vitro* [30]. In addition, LL extracts can also increase PSC survival in macrophage-parasite cocultures, which indicates that LL impairs the host protective inflammatory response by inducing AAM activation. Thioredoxin peroxidase (TPx), an antioxidant enzyme, is expressed during all developmental stages of *E. granulosus* (*s.l*.) [31]. A recent proteomic analysis identified EgTPx as one of the abundant ES proteins secreted by the parasite, and this finding suggests that this protein plays an important role in the host-parasite interactions [32–35]. In addition, we have shown that the knockdown of EgTPx gene expression by RNAi leads to impaired growth of *E. granulosus* (*sensu stricto*) both *in vitro* and *in vivo*, which indicates that the EgTPx gene plays an important role in parasite survival [36]. Previous studies have shown that the 2-Cys peroxiredoxin (Prx) derived from the flukes *S. mansoni* and *F. hepatica* can drive the activation of AAMs [13]. However, whether EgTPx is an atypical 2-Cys Prx that can induce AAMs to shape the immune response of the host to favor hydatid cyst establishment remains unclear.

In this study, we investigated the activation status of PMs in a mouse model infected with *E. granulosus* (*s.s*). larvae through intraperitoneal inoculation and evaluated the effect of an important recombinant ES product (rEgTPx) on PM activation *in vivo* and *in vitro.* Because the mTOR pathway was recently reported to play a critical role in regulating macrophage differentiation in response to helminth infection [37, 38], we further investigated whether this signaling pathway is involved in EgTPx-induced PM alternative activation.

## Methods

### Mice

Pathogen-free female BALB/c mice (6 weeks of age) were purchased from Beijing Vital River Laboratory Animal Technology Company Limited, housed in specific pathogen-free facilities with a 12 h light/dark photocycle and provided rodent chow and water *ad libitum*.

### Parasite isolation and ES products preparation

The PSCs of *E. granulosus* (*s.s.*) (genotype G1, a common sheep strain) used in this study were obtained from fertile sheep liver hydatid cysts collected from a slaughterhouse in Urumqi, Xinjiang, China, according to the protocols detailed by Zhang et al. [39]. Briefly, the PSCs were removed aseptically from intact cysts, digested with pepsin, washed several times in sterile phosphate-buffered saline (PBS) containing 100 U/ml penicillin and 100 µg/ml streptomycin and then maintained in RPMI 1640 culture medium (Gibco, Auckland, New Zealand) at 37 °C. The viability of the PSCs was determined by methylene blue exclusion analysis [40]. Only the PSC samples with higher than 95% viability were used in the study.

Parasite ES products were prepared following a reliable procedure previously described by other researchers with some modifications [29, 34]. Freshly isolated PSCs were transferred into flasks and cultured at a density of 10,000 PSCs/ml in 20 ml of sterile PBS supplemented with 100 U/ml penicillin and 100 µg/ml streptomycin at 37 °C in 5% CO_2_. Forty-eight hours after incubation, the entire volume of culture medium containing the parasite ES products (20 ml) was collected, concentrated 40-fold using Amicon Ultra-15 centrifugal filters (Millipore, Billerica, MA, USA), and filtered through a 0.22 µm filter (Millipore, Billerica, MA, USA). The concentration of the ES products was measured using a BCA assay (Thermo Fisher Scientific, Rockford, IL, USA), and the products were stored at − 80 °C until use.

### Preparation of a recombinant ES product (rEgTPx) and its variant (rvEgTPx)

Total RNA from *E. granulosus* (*s.s.*) PSCs was isolated using TRIzol reagent (Invitrogen, Carlsbad, CA, USA) according to the manufacturer’s recommended protocol. The extracted RNA was treated with RNase-free DNase I (Fermentas, Vilnius, Lithuania) to remove potential genomic DNA contaminants and then reverse transcribed into cDNA using the PrimeScript^TM^ RT Reagent Kit (Fermentas, Vilnius, Lithuania). The complete open reading frame (ORF) of EgTPx was amplified using gene-specific primers containing *Eco*RI and *Not* I restriction sites and ligated into the pET-28a vector with an N-terminal 6× His-tag (Novagen, Madison, WI, USA). The expression construct was transformed into competent *Escherichia coli* BL21 (DE3) cells (Tiangen, Beijing, China.) and purified using a His-binding resin (Novagen) according to the manufacturer’s instructions. A recombinant variant of EgTPx (rvEgTPx) was prepared by synthesizing the gene with the reactive Cys48 and Cys169 residues replaced by Gly residues. Residual bacterial endotoxin was removed from the purified recombinant proteins by phase separation using Triton X-114. The protein purity was analyzed by sodium dodecyl sulfate-polyacrylamide gel electrophoresis (SDS-PAGE), and the protein concentrations were measured using a BCA protein assay kit (Thermo Fisher Scientific).

The specific enzymatic activities of rEgTPx and rvEgTPx were determined through metal-catalyzed oxidation (MCO) DNA cleavage protection assays [31]. Briefly, purified rEgTPx and rvEgTPx proteins with final concentrations ranging from 6.25 to 100 μg/ml were incubated in 50 μl reaction mixtures containing 16.5 μM FeCl_3_ and 3.3 mM dithiothreitol (DTT) for 2 h at 37 °C and then with pET28a (800 ng) supercoiled plasmid DNA for an additional 2.5 h. The degree of DNA degradation was evaluated by electrophoresis with a 1.0% (w/v) agarose gel. The correct folding of rEgTPx and its variant was confirmed by assessing their migration *via* SDS-PAGE under reducing and nonreducing conditions [13].

### Animal infection and treatment with parasite antigens

For infection, each mouse was intraperitoneally (i.p.) transplanted with 50 *E. granulosus* (*s.s.*) microcysts (250–300 μm in diameter) cultured *in vitro* as previously described [39] or directly inoculated with 200 μl of a suspension containing 2000 live PSCs in PBS [29]. The control mice were injected with 200 μl of PBS. After the experimental period (3 or 6 months post-infection), the mice were necropsied, and peritoneal exudate cells (PECs) were harvested through three washes of the PerC with 5 ml of sterile PBS. For parasite antigen treatment, the mice were administered nine i.p. injections of 5 μg of the parasite ES products or purified rEgTPx or rvEgTPx on alternate days, and PBS was injected as a negative control. PECs were harvested 2 days after the final injection.

### *In vitro* treatment of PMs with parasite antigens

For *in vitro* experiments, PMs were elicited through the i.p. injection of 800 μl of 4% w/v sterile thioglycollate medium into mice and the harvesting of PECs four days post-injection [12]. For antigen treatment, the purified PMs were adjusted to a density of 1 × 10^6^ cells/ml and then cultured with PBS or 10 μg/ml parasite ES products, rEgTPx or rvEgTPx in a six-well plate for 24 h, and lipopolysaccharide (LPS; 50 ng/ml, Sigma, St. Louis, MO, USA) and IL-4 (20 ng/ml, PeproTech, Rocky Hill, CT, USA) were used as the negative and positive controls, respectively [19]. The cells were washed with PBS and then analyzed by quantitative real-time PCR (qRT-PCR).

To determine whether the PI3K/AKT/mTOR pathway is involved in the alternative activation of PMs, the PMs were preincubated with the inhibitors LY294002 (an Akt-specific inhibitor, Selleck Chemicals, Houston, TX, USA) and rapamycin (a mTOR inhibitor, Selleck Chemicals) for 1 h and then cultured with parasite antigens (50 μg/ml ES products and 10 μg/ml rEgTPx) for 12 h. After the treatments, cell lysates were collected for assessment of the Akt and mTOR phosphorylation levels by Western blotting.

### Flow cytometry analysis

PMs were purified from PECs as described previously [12], and the PEC purity was analyzed by fluorescence-activated cell sorting (FACS) staining using the macrophage markers F4/80 and CD11b. For cell morphology analysis, adherent macrophages from uninfected and *E. granulosus* (*s.s.*)-infected mice were imaged using an inverted microscope (Leica, Wetzlar, Germany). For FACS analysis, the PECs were adjusted to a density of 1 × 10^6^ cells/ml, incubated with anti-CD16/CD32 antibodies (BioLegend, San Diego, CA, USA) for 20 min at 4 °C in the dark and then stained with the following fluorescently labeled antibodies specific for cell surface markers for 25 min: anti-CD45; anti-NK1.1; anti-CD3; anti-CD19; anti-F4/80; anti-CD11b, anti-CD80; anti-CD86; and anti-MHC II (BioLegend). For detection of the CAM and AAM phenotypes, the PECs were stained with anti-CD45, anti-NK1.1, anti-CD3, anti-CD19, anti-F4/80, and anti-CD11b antibodies at 4 °C for 30 min, washed and then fixed in Cytofix/Cytoperm (BD Biosciences, Franklin Lakes, NJ, USA) according to the manufacturer’s instructions for staining with anti-CD206 (AAM marker) and anti-iNOS (CAM marker) antibodies. All samples were run on an LSRFortessa flow cytometer (BD Immunocytometry Systems, San Jose, CA, USA), and the data were analyzed using FlowJo software (version V10; TreeStar Inc., Ashland, OR, USA). Corresponding fluorochrome-labeled IgG isotype control antibodies were used in parallel. The percentage of cells that were stained positive for each surface protein was determined by comparing the test samples with the isotype control-stained samples. Information on the antibodies utilized in this assay is shown in Additional file 1: Table S1.

### Western blot analysis

The cells were lysed in RIPA buffer containing a phosphatase and protease inhibitor cocktail (EMD Millipore, Temecula, CA, USA). Thirty micrograms of protein was separated by 10% SDS-PAGE, transferred to polyvinylidene fluoride (PVDF) membranes (Millipore Corp., MA, USA), and incubated with rabbit anti-phospho-mTOR (1:1000), anti-mTOR (1:1000), anti-phospho-AKT (1:1000), anti-AKT (1:1000), anti-PI3K (1:1000), or anti-β-actin (1:1000) antibodies overnight at 4 °C. The membranes were then incubated with alkaline phosphatase-conjugated anti-rabbit IgG antibodies (1:2000; Cell Signaling Technology, Danvers, MA, USA) for 1 h and visualized using a BCIP/NBT kit (Invitrogen, Carlsbad, CA, USA).

### qRT-PCR analysis

Total RNA from PMs was isolated and reverse transcribed into cDNA as described above. qRT-PCR was conducted using the SYBR Green PCR premix (TaKaRa, Dalian, China) and run on a qRT-PCR instrument (iQ5 Bio-Rad, Hercules, CA, USA) as described previously [41]. The reaction conditions were as follows: stage 1, 95 °C for 30 s; stage 2, 40 cycles of 95 °C for 5 s and 60 °C for 30 s; and stage 3, melting curve analysis. The relative expression of the target genes was determined by the comparative quantification cycle (Cq) normalized against the housekeeping gene (GAPDH) using the 2^−ΔΔCq^ method [29, 42]. The sequences of all primers used in this analysis are shown in Table 1.

**Table 1:** Sequences of the qRT-PCR primers

Gene	GenBank ID	Forward primer	Reverse primer
iNOS	NM_010927.3	TTCACCCAGTTGTGCATCGACCTA	TCCATGGTCACCTCCAACACAAGA
TNF-α	NM_013693.3	AAGCCTGTAGCCCACGTCGTA	AGGTACAACCCATCGGCTGG
IL-10	NM_010548.2	GCCAGAGCCACATGCTCCTA	GATAAGGCTTGGCAACCCAAGTAA
Ym1	NM_009892.3	TCTCTACTCCTCAGAACCGTCAGA	GATGTTTGTCCTTAGGAGGGCTTC
Fizz1	NM_181596.4	TACTTGCAACTGCCTGTGCTTACT	TATCAAAGCTGGGTTCTCCACCTC
Arg1	NM_007482.3	CTCCAAGCCAAAGTCCTTAGAG	AGGAGCTGTCATTAGGGACATC
GAPDH	NM_001289726.1	CATGGCCTTCCGTGTTCCTA	CCTGCTTCACCACCTTCTTGAT

### Statistical analysis

The results are presented as the mean ± standard error of the mean (SEM) and were analyzed using GraphPad Prism software (GraphPad Software, Inc., USA). The statistical significance was assessed by one-way ANOVA with Tukey’s multiple comparison test, and Student’s t-test was used for the comparisons of only two groups. Differences were considered significant at *P < 0.05, **P < 0.01, ***P < 0.001, ****P < 0.0001.

## Results

### *Echinococcus granulosus (s.s.)* infection induces PMs to differentiate into an alternatively activated phenotype

**Fig. 1:**
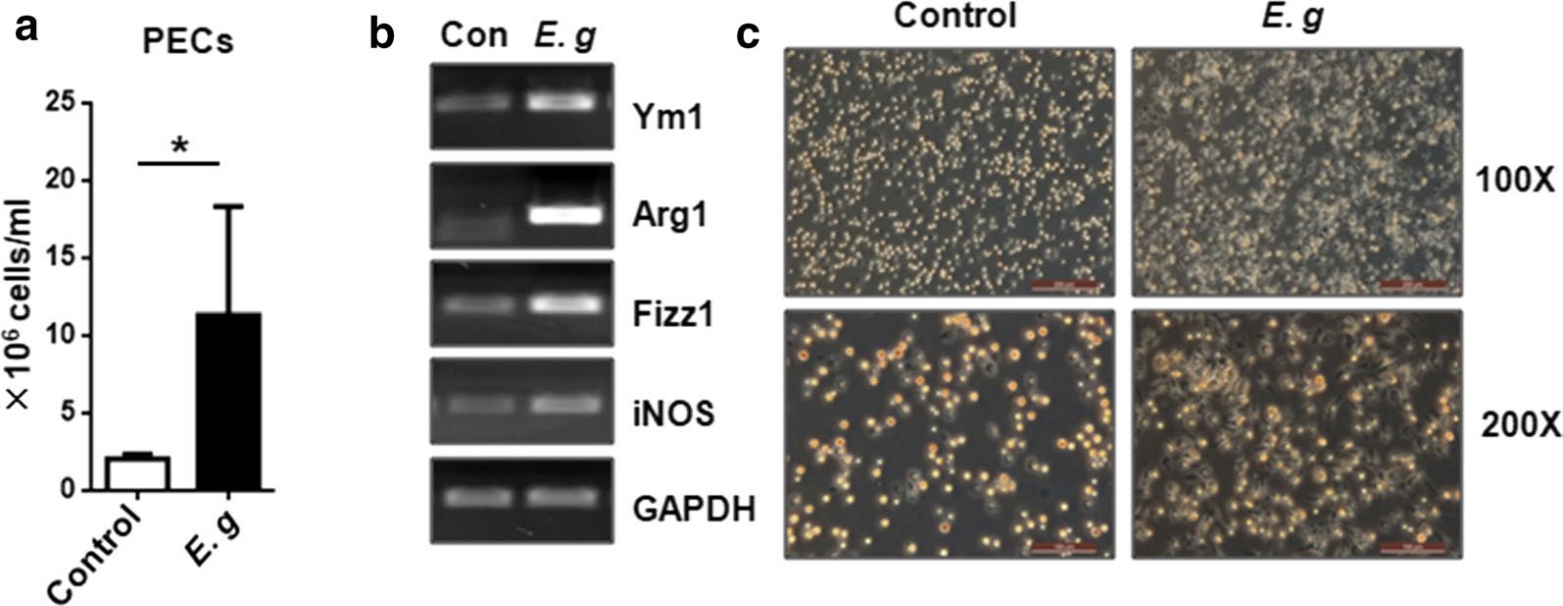
*E. granulosus* (*s.s.*) infection induces an alternatively activated phenotype in macrophages and different cell morphologies. **a** Total counts of peritoneal exudate cells (PECs) isolated from *E. granulosus*-infected or control mice. **b** Expression levels of phenotypic markers (Ym1, Arg1, Fizz1 and iNOS) in adherent PECs isolated from control mice and infected mice analyzed by RT-PCR. **c** Morphological observation of adherent PECs isolated from control mice and infected mice. The data are shown as the mean ± SEM (*n* = 5). *Abbreviations*: Con, control; *E. g*., *E. granulosus* (*s.s.*). *P < 0.05. *Scale-bars*: **c**, 200 µm for 100×; 100 µm for 200×

To determine whether *E. granulosus* (*s.s.*) infection induces the alternative activation of PMs, PMs were collected by adherence from mice 6 months after infection with *E. granulosus* (*s.s.*) microcysts. The expression of the genes encoding Ym1, Fizz1 and Arg1 (AAM marker) and iNOS (CAM marker) in PMs was examined. We found that *E. granulosus* (*s.s.*) infection increased the recruitment of PECs to the PerC (*t*_(8)_ = 2.65, *P* = 0.03) (Fig. 1a). PMs isolated from the PerC of infected mice (Eg-Mφ) showed higher expression levels of Ym1, Fizz1 and Arg1 than PMs isolated from control mice treated with PBS (PBS-Mφ). Very low levels of iNOS expression were detected (Fig. 1b). In addition, the morphology of the Eg-Mφ differed from that of the PBS-Mφ. The Eg-Mφ were tightly adherent with spread-out processes, whereas the PBS-Mφ were less adherent and showed a more rounded shape, which might indicate a distinct functional role for Eg-Mφ (Fig. 1c).

### rEgTPx induces PMs to differentiate into an alternatively activated phenotype *in vivo*

To evaluate the effects of the important ES product EgTPx on the activation of PMs and to determine whether the antioxidant activity of EgTPx is involved in PM activation, we first constructed rEgTPx and then created an inactive recombinant variant (rvEgTPx) by replacing Cys48 and Cys169 with Gly residues (Additional file 2: Figure S1a). At a relatively high concentration, rEgTPx prevented the processing of plasmid DNA from a supercoiled form to a nicked form in an MCO system. However, rvEgTPx did not protect against this type of damage (Additional file 2: Figure S1b). In addition, rvEgTPx did not form disulfide linkages and thus mainly remained in its monomeric form under nonreducing conditions (Additional file 2: Figure S1c), which further confirmed that the variant was constructed successfully.

**Fig. 2:**
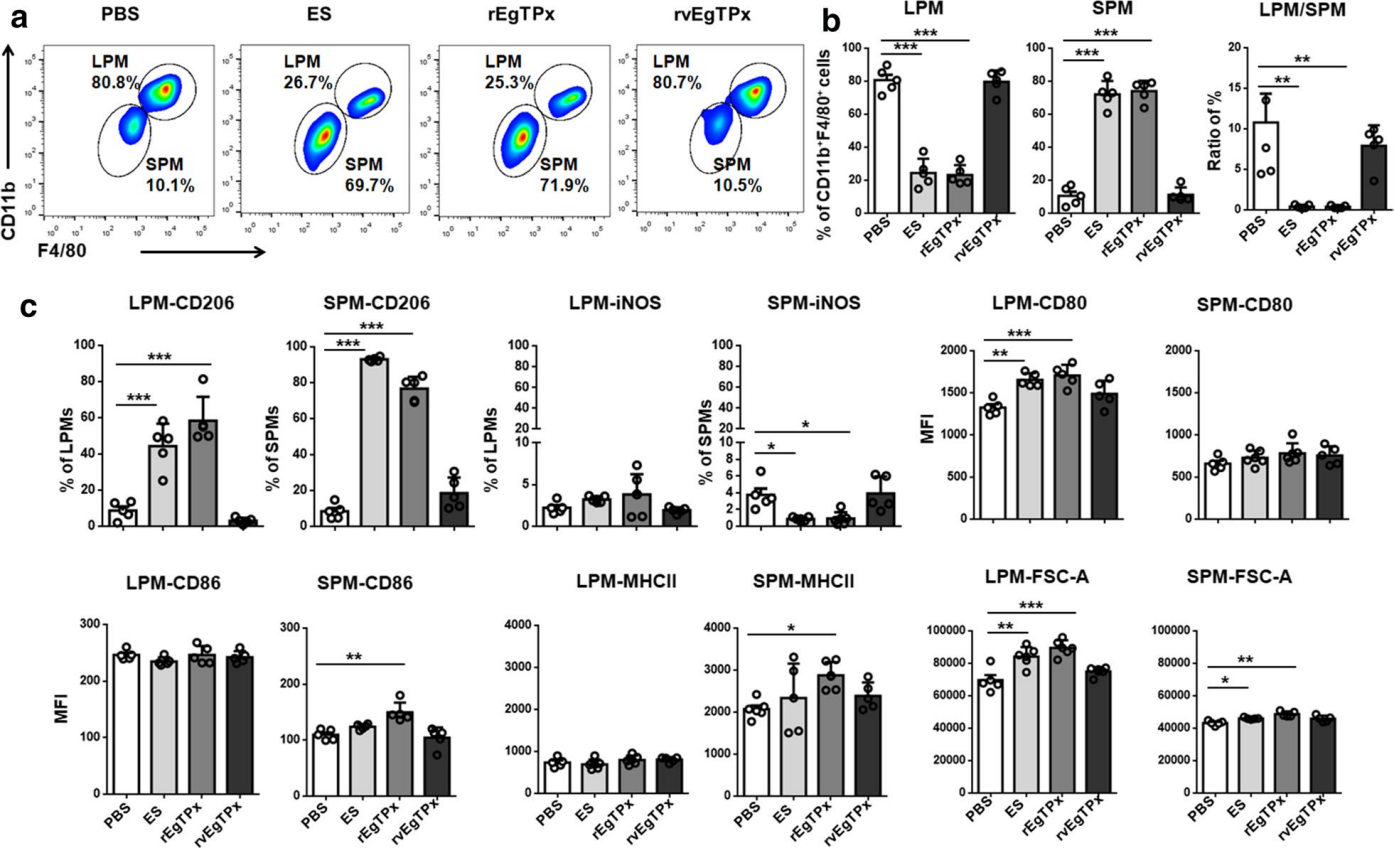
Parasite antigen treatment skewed the PM subsets and induced an alternatively activated phenotype in PMs *in vivo*. **a** Representative FACS plots of LPMs and SPMs. **b** Percentages of CD11b^high^F4/80^high^ LPMs and CD11b^int^F4/80^int^ SPMs in the peritoneal exudates from mice belonging to the different groups. **c** Percentages of iNOS^+^ macrophages (CAMs) and CD206^+^ macrophages (AAMs) in LPMs and SPMs and the expression patterns of cell surface markers (CD80, CD86, and MHCII) in LPMs and SPMs after parasite antigen treatment. The sizes of LPMs and SPMs were determined using the FSC-A parameter. The data are depicted as the mean fluorescence intensities (MFIs) for each surface marker. The data are shown as the mean ± SEM (*n* = 5). *P < 0.05, **P < 0.01, ***P < 0.001

**Fig. 3:**
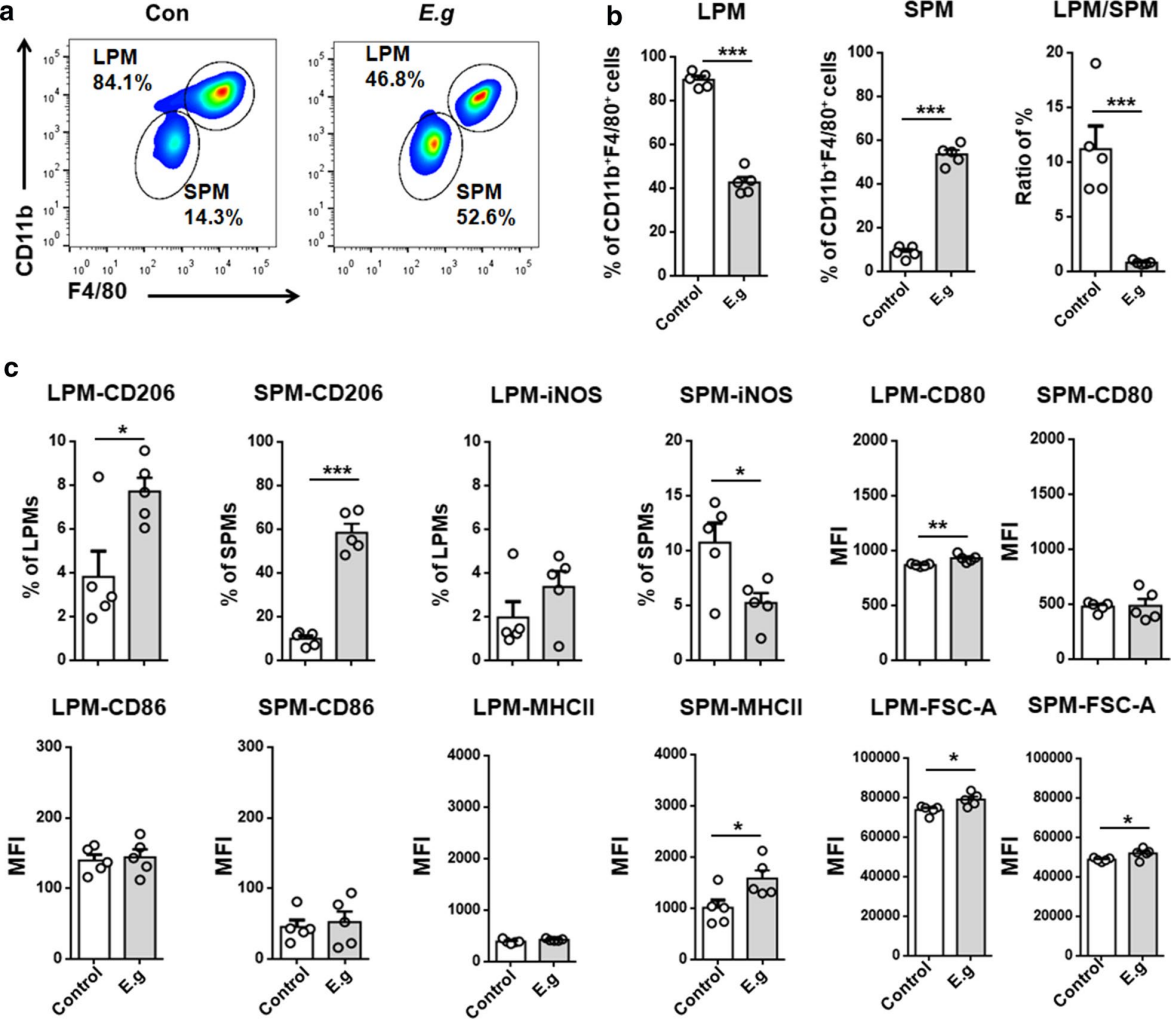
*E. granulosus* (*s.s.*) infection skewed the PM subsets and induced an alternatively activated phenotype in PMs. **a** Representative FACS plots of LPMs and SPMs. **b** Percentages of CD11b^high^F4/80^high^ LPMs and CD11b^int^F4/80^int^ SPMs in the peritoneal exudates from infected mice and control mice. **c** Percentages of iNOS^+^ macrophages (CAMs) and CD206^+^ macrophages (AAMs) in LPMs and SPMs and the expression patterns of cell surface markers (CD80, CD86, and MHCII) in LPMs and SPMs after *E. granulosus* (*s.s.*) infection. The sizes of LPMs and SPMs were determined using the FSC-A parameter. The data are depicted as the mean fluorescence intensities (MFIs) for each surface marker and are shown as the mean ± SEM (*n* = 5). *P < 0.05, **P < 0.01, ***P < 0.001. *Abbreviation*: *E. g*., *E. granulosus* (*s.s.*)

Mice were then administered nine intraperitoneal injections of rEgTPx, rvEgTPx or native parasite ES products and were also intraperitoneally injected with PSCs for 3 months in parallel with the antigen treatments. PECs were harvested and examined by RT-PCR and FACS. Similar to the results obtained with *E. granulosus* (*s.s.*) infection, PMs isolated from ES- and rEgTPx-treated mice also showed higher expression levels of AAM-related genes (Ym1, Fizz1 and Arg1) than these cells from the control mice (Additional file 2: Figure S1d). We identified the CD11b^high^F4/80^high^ LPM and CD11b^low^F4/80^low^ SPM populations in the PECs after excluding cell aggregates and other peritoneal immune cells using an antibody cocktail that recognizes CD3, NK1.1 and CD19 (Additional file 3: Figure S2). The percentages of LPMs and SPMs were significantly decreased (*F*_(3, 16)_ = 100.4, *P* < 0.0001) and increased (*F*_(3, 16)_ = 164.5, *P* < 0.0001) in the ES and rEgTPx treatment groups and the *E. granulosus* (*s.s.*) infection group compared with the rvEgTPx and PBS control groups (Figs. 2a, b, 3a, b). We subsequently examined the expression of CAM (iNOS) and AAM (CD206) markers in these PM subsets, and the results showed that compared with the administration of PBS, the delivery of ES products and rEgTPx or infection with PSCs significantly increased the percentage of CD206^+^ macrophages in both the LPM (*F*_(3 16)_ = 41.22, *P* < 0.0001) and SPM (*F*_(3, 16)_ = 251.7, *P* < 0.0001) subsets and reduced the percentage of iNOS^+^ macrophages in the SPM subset (*F*_(3, 16)_ = 8.9, *P* = 0.0008) (Figs. 2c, 3c and Additional file 4: Figure S3). Interestingly, lack of EgTPx enzymatic activity (rvEgTPx delivery) had no effect on the ability to skew PMs toward the AAM phenotype. In addition, we found that the mean fluorescence intensity (MFI) of CD80 in the LPMs from ES- and rEgTPx-injected and PSC-infected mice was significantly increased (*F*_(3, 16)_ = 10.99, *P* = 0.0004), but no differences in the MFIs of CD86 and MHC II were detected. In contrast, the MFIs of MHC II were significantly increased (*F*_(3, 16)_ = 3.19, *P* = 0.0486) in the SPMs from rEgTPx-injected and PSC-infected mice. The MFI of FSC-A in both the LPMs (*F*_(3, 16)_ = 16.03, *P* < 0.0001) and SPMs (*F*_(3, 16)_ = 11.72, *P* = 0.0003) from ES- and rEgTPx-injected and PSC-infected mice was higher than that in the same cell populations from control mice (Figs. 2c, 3c).

### rEgTPx induces PMs to differentiate into an alternatively activated phenotype *in vitro*

**Fig. 4:**
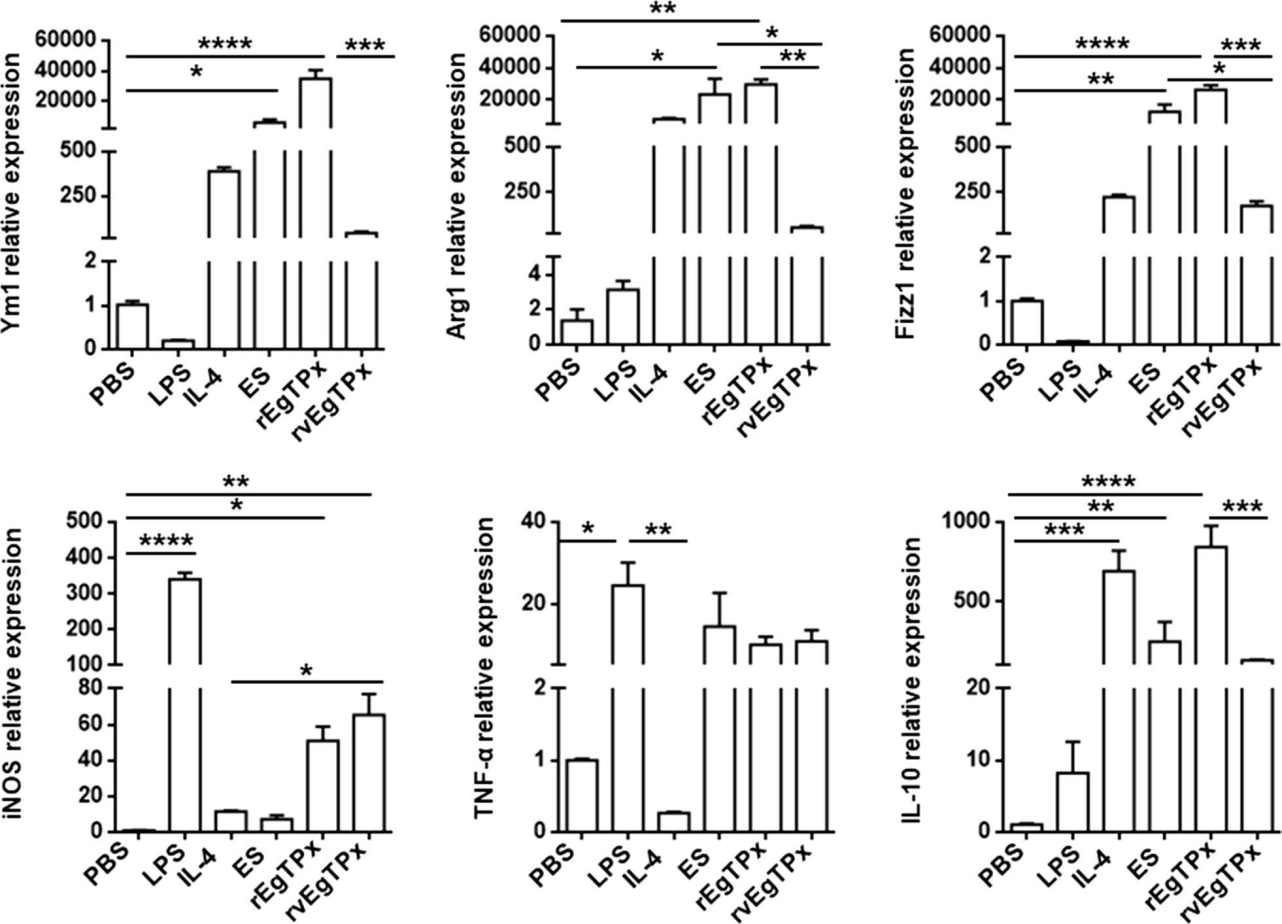
Parasite antigen treatment induced an alternatively activated phenotype in PMs *in vitro*. PMs from normal mice were purified and stimulated with different parasite antigens for 24 h. LPS and IL-4 were included as positive controls for CAMs and AAMs, respectively. The transcript levels of AAM markers (YM1, Arg1, Fizz1 and IL-10) and CAM markers (iNOS and TNF-α) were evaluated by real-time PCR. The expression levels of each molecule were determined by the comparative Cq value normalized against the GAPDH using the 2^−ΔΔCq^ method. The data are presented as the mean ± SEM from three independent experiments. *P < 0.05, **P < 0.01, ***P < 0.001, ****P < 0.0001

To evaluate the effects of rEgTPx on macrophage phenotypes *in vitro*, thioglycollate-elicited PMs were purified and treated with ES products, rEgTPx, rvEgTPx, LPS (negative control) or IL-4 (positive control), and the expression of CAM and AAM markers in these PMs were then determined by qRT-PCR. The results showed that compared with the PBS control treatment, the ES product and rEgTPx treatments significantly upregulated the expression of Ym1 (*F*_(5, 12)_ = 38.92, *P* < 0.0001), Fizz1 (*F*_(5, 12)_ = 22.74, *P* < 0.0001), Arg1 (*F*_(5, 12)_ = 9.50, *P* = 0.0001) and the level of the anti-inflammatory cytokine IL-10 (*F*_(5, 12)_ = 15.56, *P* < 0.0001). However, the expression of iNOS and TNF-α was detectable but relatively weak. In addition, treatment with rvEgTPx also increased the expression of Ym1, Fizz1, and Arg1, but the detected levels were lower relative to those observed after treatment with the ES product or rEgTPx (Fig. 4). These results suggest that ES and rEgTPx can preferentially induce an anti-inflammatory AAM phenotype *in vitro*.

### rEgTPx induces AAMs by activating the PI3K/AKT/mTOR signaling pathway

**Fig. 5:**
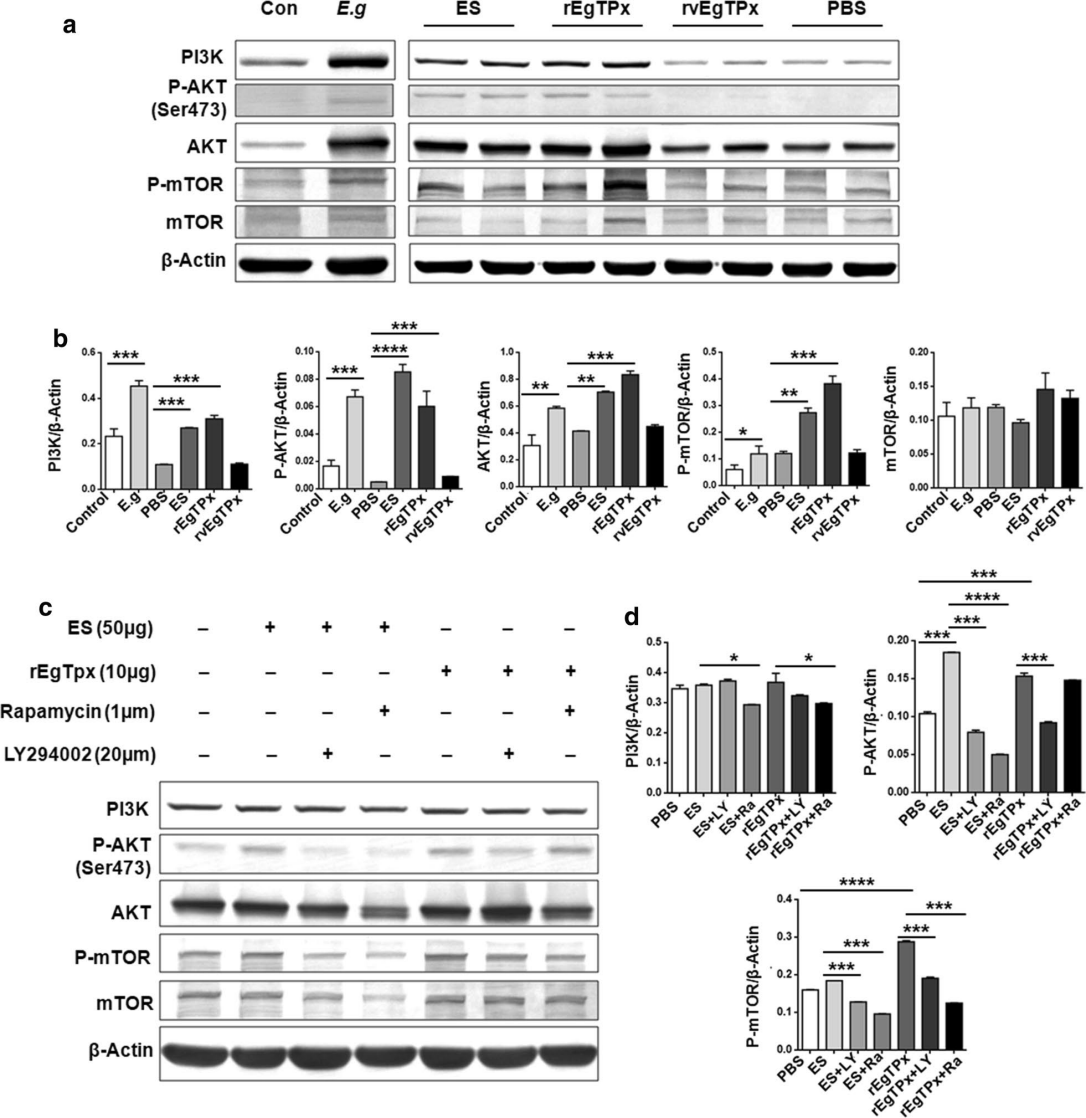
Parasite antigen (rEgTPx) induces an alternatively activated phenotype in macrophages *via* the PI3K/AKT/mTOR pathway. **a, b** Western blot analysis and quantification of phosphorylated and total mTOR and AKT protein levels in whole-cell lysates of adherent PECs isolated from control mice and parasite-infected and antigen-treated mice. **c**, **d** Effects of inhibitors on P-AKT and P-mTOR expression in macrophages. The data are shown as the mean ± SEM (*n* = 5). *P < 0.05, **P < 0.01, ***P < 0.001, ****P < 0.0001. *Abbreviations*: Con, control; *E. g*., *E. granulosus* (*s.s.*)

To determine whether the PI3K/AKT/mTOR signaling pathway is involved in the parasite antigen-induced alternative activation of macrophages, the phosphorylation status of mTOR pathway components in PMs isolated from the parasite-infected and antigen-injected mice described above was assessed through a Western blot analysis. The ES product and rEgTPx injections induced higher phosphorylation levels of mTOR (*F*_(5, 24)_ = 36.02, *P* < 0.0001) and Akt (*F*_(5, 24)_ = 36.21, *P* < 0.0001) in the PMs compared with those obtained after PBS injection, and these results were consistent with those observed after *E. granulosus* (*s.s.*) infection. Similarly, the expression of PI3K and AKT was also upregulated. However, the phosphorylation levels of mTOR and AKT in the PMs derived from the rvEgTPx-injected mice were similar to those in the PMs derived from the PBS control mice, and the expression of PI3K was also similar (Fig. 5a, b, Additional file 5: Figure S4).

We used various inhibitors in this study to further identify the importance of this pathway in the parasite antigen-induced alternative activation of macrophages. We found that LY294002 (an AKT-specific inhibitor) and rapamycin (an mTOR inhibitor) significantly attenuated the phosphorylation of AKT (*F*_(6, 14)_ = 456.4, *P* < 0.0001) and mTOR (*F*_(6, 14)_ = 1428, *P* < 0.0001) compared with the levels found in the ES product- or rEgTPx-stimulated PMs (Fig. 5c, d, Additional file 5: Figure S4). These results indicate that the parasite antigen-induced alternative activation of PMs is likely mediated by the PI3K/AKT/mTOR signaling pathway.

## Discussion

An accumulating body of evidence shows that the alternative activation of macrophages is a hallmark of helminth infections and plays an important immunomodulatory role in these infections by not only limiting inflammation but also preventing excessive tissue remodeling [15, 18, 43, 44]. This limited inflammation has been confirmed as one of the main survival strategies of helminths that allows them to reside in their intermediate hosts for a long time [45]. However, the activation status of macrophages during infection with *E. granulosus* (*s.s.*), which is a major species of the genus *Echinococcus* belonging to the family *Taeniidae* of the cestode platyhelminths [46, 47] that causes CE, remains poorly defined. In this study, we showed that intraperitoneal infection with *E. granulosus* (*s.s.*) larvae and the injection of ES products secreted by the parasite, including EgTPx, induced the recruitment of large numbers of PMs to the PerC and the preferentially differentiation of PMs toward an AAM phenotype. Furthermore, we found that EgTPx induces the alternative activation of PMs *via* the PI3K/AKT/mTOR pathway and that this activation is dependent on the antioxidant activity of EgTPx, which suggests that EgTPx plays multiple roles in favoring the survival of hydatid cysts in the host.

The mouse model of intraperitoneal infection with *E. granulosus* (*s.l*.) is the most widely used model that mirrors the secondary infection that occurs in the intermediate host after fertile cyst rupture [4, 48], and PMs, which are one of the most-studied macrophage populations [9, 10], have been used in many recent studies investigating the responses of macrophages related to parasitic diseases such as schistosomiasis [18, 19]. Therefore, we characterized the phenotypes of PMs isolated from the PerC of infected mice. Similar to the findings obtained with other helminth infections [13, 17, 18], our results showed that *E. granulosus* (*s.s.*) infection recruited a relatively large number of PECs to the PerC and that most of the recruited cells were preferentially differentiated into the AAM phenotype during the late stage of *E. granulosus* (*s.s.*) infection. Previous studies using this model have shown that macrophages are also prominent in the early infiltrate, but that infiltration does not develop into a severe inflammatory response during the cyst establishment stage. Thus, based on our results, we speculate that the parasite might have adapted to avoid the host inflammatory response through the activation of AAMs.

Recent studies have shown that the LL, the interface between the parasite and the host, is permeable, which allows *E. granulosus* (*s.l*.) ES products to pass through and directly interact with the immune cells around the cyst [32, 49]. It is evident that LL extracts, which might contain some uncharacterized ES products, can induce arginase expression *in vitro* and enhance the induction of Ym1 expression *in vivo*, which indicates that the LL and the materials shed from the LL could have the capacity to promote AAM activation to favor parasite survival [30, 50, 51]. Our present results showed that EgTPx, one of the abundant ES products that has been confirmed to play an important role in antioxidant defense against the host during development [36], could not only induce the alternative activation of PMs *in vivo* but also modulate the differentiation of PMs toward an alternatively activated phenotype *in vitro.* These results are consistent with those found for Prx derived from the flukes *S. mansoni* and *F. hepatica* [13].

In addition, infectious and inflammatory stimuli, such as *Trypanosoma cruzi* infection, usually alter the PM composition in the PerC due to the disappearance of LPMs of embryogenic precursor origin and the notable increase in the numbers of SPMs of bone marrow origin, which results in marked LPM disappearance and SPM expansion in the PerC [9–11]. We and other research groups have also shown that LL-derived particles and rEgTPx induce similar changes that skew the PM subsets from LPMs to SPMs [51]. SPMs generally present a proinflammatory functional profile during inflammation initiation and might elicit acute inflammation. However, we found that the percentages of CD206^+^ SPMs and CD206^+^ LPMs in mice were significantly increased after the administration of nine injections of rEgTPx or ES products. These results indicate that rEgTPx and ES products derived from *E. granulosus* (*s.s.*) can not only recruit a wave of blood monocytes into the PerC that differentiate into SPMs but also skew the differentiation of SPMs toward an anti-inflammatory phenotype over time, which would favor the establishment of parasites in the host and, to some extent, mimic natural infection.

Our analysis of surface markers showed that SPMs, particularly those from rEgTPx-injected mice, expressed high levels of MHC-II, whereas LPMs did not express this classical activation marker but did exhibit higher expression levels of CD80 than those found in SPMs. These observations are consistent with the reported characteristics of these two types of cells [10]. Furthermore, macrophages reportedly have the ability to promote immune tolerance by directly inducing Tregs [52], and we detected a predominant upregulation of the mRNA level of IL-10, a crucial Treg-inducing cytokine [53], in PMs exposed to rEgTPx *in vitro*, which might indicate that these PMs support Treg generation. To further clarify whether this immunoregulatory function of EgTPx can be attributed to its antioxidant activity, we also constructed an inactive variant of EgTPx and revealed that the EgTPx-mediated alternative activation of macrophages depends on the antioxidant activity of the enzyme.

Various lines of evidence show that mTOR and its related pathways can regulate the functions of dendritic cells [54, 55], monocytes [56] and macrophages [38, 57]. In addition, recent studies have clearly demonstrated that AKT and mTOR signaling play key roles in promoting the alternative activation of macrophages [37, 58]. Here, we found that rEgTPx and ES products secreted by *E. granulosus* (*s.s.*) trigger AKT and mTOR phosphorylation and upregulate the expression of the upstream regulator PI3K, which indicates that stimulation with these parasite antigens activates the PI3K/AKT/mTOR pathway. However, this activated pathway can be suppressed by pre-treatment with an inhibitor of AKT (LY294002) or mTOR (rapamycin). Therefore, the activation of the PI3K/AKT/mTOR pathway is, at least in part, the most likely mechanistic explanation for the effects of rEgTPx and ES products on the alternative activation of macrophage. Inhibition of the mTOR signaling pathway might become a novel therapeutic approach for altering the survival of parasites.

## Conclusions

Our data demonstrate that PMs are recruited and preferentially induced to differentiate toward an alternatively activated phenotype after intraperitoneal infection with *E. granulosus* (*s.s.*) larvae. EgTPx, an important antioxidant enzyme secreted by *E. granulosus* (*s.s.*), can induce the alternative activation of PMs both *in vivo* and *in vitro*, and this induction is dependent on the antioxidant activity of this enzyme, which suggests that EgTPx plays dual roles in the survival of cysts in the host by not only resisting oxidative damage but also regulating macrophage activation to overcome inflammation. Furthermore, we found that the PI3K/AKT/mTOR signaling pathway might play an important role during *E. granulosus* (*s.s.*) antigen (EgTPx)-induced alternative activation of macrophages, which implies that inhibition of the mTOR pathway to modulate the macrophage activation status could become a novel therapeutic strategy for controlling parasitic diseases and some inflammatory disorders. Further identification of the immunomodulatory function of the important ES product EgTPx will promote the development of novel preventive strategies against CE.

## Supplementary information

**Additional file 1: Table S1.** Antibodies utilized in the flow cytometry analysis.

**Additional file 2: Figure S1.** Functional expression and characterization of recombinant EgTPx. **a** Multiple sequence alignment of EgTPx (AAL84833), *E. multilocularis* TPx (EmTPx; BAC11863), mouse Prx2 (MsPrx2; Q61171), human Prx2 (HsPrx2; P32119), *Rattus* Prx2 (RatPrx2; NP_058865), *Ovis aries* Prx2 (OvisPrx2; NP_001159672), *Brugia malayi* TPx (BmTPx; Q17172) and *Taenia multiceps* TPx (TmTPx; ADW77118). The arrows indicate the redox-active Cys48 and Cys169 residues that were replaced by Gly to generate EgTPx variants (rvEgTPx). **b** Protection of plasmid DNA from oxidative damage by rEgTPx in MCO system. M: DNA marker; Lane 1: DNA alone; Lane 2: DNA in water with incubation; Lane 3: DNA in MCO system with incubation; Lane 4: DNA in MCO system incubated with BSA; Lanes 5–9 and 10–14: DNA in MCO system incubated with 400, 200, 100, 25 and 12.5 μg/ml of rEgTPx and rvEgTPx, respectively; NF, nicked form of the plasmid; SF, supercoiled form of the plasmid. **c** rEgTPx and rvEgTPx were electrophoresed under reducing (with DTT) and nonreducing conditions (without DTT). The monomeric and dimeric forms of the enzymes are indicated by arrows. **d** RT-PCR was used to assess the expression of Ym1, Arg1, Fizz1 and iNOS in PMs isolated from *E. granulosus* (*s.s.*)-infected and parasite antigen-treated mice.

**Additional file 3: Figure S2.** Gating strategy used for the identification of large and small peritoneal macrophages (LPMs and SPM). LPMs: CD45^+^CD3^−^CD19^−^NK1.1^−^ and CD11b^high^F4/80^high^. SPMs: CD45^+^CD3^−^CD19^−^NK1.1^−^ and CD11b^low^F4/80^low^.

**Additional file 4: Figure S3.** Representative FACS plots gated on PM subsets from *E. granulosus* (*s.s.*)-infected and parasite antigen-treated mice. **a, b** Intracellular staining of iNOS^+^ and CD206^+^ in LPMs and SPMs, respectively.

**Additional file 5: Figure S4.** Uncropped Western blots corresponding to the results shown in Fig. 5.

## Data Availability

The datasets supporting the conclusions of this article are included within the article and its additional files.

## Abbreviations

AAMs or M2: alternatively activated macrophages
Arg-1: arginase-1
CAMs or M1: classically activated macrophages
ES products: excretory-secretory products
EgTPx: *Echinococcus granulosus* thioredoxin peroxidase
FACS: fluorescence-activated cell sorting
iNOS: inducible nitric oxide synthase
LPMs: large peritoneal macrophages
MFI: mean fluorescence intensity
NO: nitric oxide
PSCs: protoscoleces
PBS: phosphate-buffered saline
PECs: peritoneal exudate cells
PMs: peritoneal macrophages
rEgTPx: recombinant EgTPx
rvEgTPx: recombinant variant EgTPx
SPMs: small peritoneal macrophages

## References

[1] Wen H, Vuitton L, Tuxun T, Li J, Vuitton DA, Zhang W (2019). Echinococcosis: advances in the 21st century. Clin Microbiol Rev..

[2] Zhang W, Li J, McManus DP (2003). Concepts in immunology and diagnosis of hydatid disease. Clin Microbiol Rev..

[3] Vatankhah A, Halasz J, Piurko V, Barbai T, Raso E, Timar J (2015). Characterization of the inflammatory cell infiltrate and expression of costimulatory molecules in chronic *Echinococcus granulosus* infection of the human liver. BMC Infect Dis..

[4] Mourglia-Ettlin G, Merlino A, Capurro R, Dematteis S (2016). Susceptibility and resistance to *Echinococcus granulosus* infection: associations between mouse strains and early peritoneal immune responses. Immunobiology..

[5] Fotiadis C, Sergiou C, Kyrou I, Troupis TG, Tselentis J, Doussaitou P (1999). Experimental *echinococcus* infection in the mouse model: pericystic cellular immunity reaction and effects on the lymphoid organs of immunocompetent and thymectomized mice. Vivo..

[6] Magambo JK, Zeyhle, Wachira TM, Wachira J, Raasen T (1995). Cellular immunity to *Echinococcus granulosus* cysts. Afr J Health Sci..

[7] Zhang W, Ross AG, McManus DP (2008). Mechanisms of immunity in hydatid disease: implications for vaccine development. J Immunol..

[8] Muraille E, Leo O, Moser M (2014). TH1/TH2 paradigm extended: macrophage polarization as an unappreciated pathogen-driven escape mechanism?. Front Immunol..

[9] Cassado Ados A, D’Imperio Lima MR, Bortoluci KR (2015). Revisiting mouse peritoneal macrophages: heterogeneity, development, and function. Front Immunol..

[10] Ghosn EE, Cassado AA, Govoni GR, Fukuhara T, Yang Y, Monack DM (2010). Two physically, functionally, and developmentally distinct peritoneal macrophage subsets. Proc Natl Acad Sci USA.

[11] Cassado Ados A, de Albuquerque JA, Sardinha LR, Buzzo Cde L, Faustino L, Nascimento R (2011). Cellular renewal and improvement of local cell effector activity in peritoneal cavity in response to infectious stimuli. PLoS ONE.

[12] Nair MG, Cochrane DW, Allen JE (2003). Macrophages in chronic type 2 inflammation have a novel phenotype characterized by the abundant expression of Ym1 and Fizz1 that can be partly replicated *in vitro*. Immunol Lett..

[13] Donnelly S, Stack CM, O’Neill SM, Sayed AA, Williams DL, Dalton JP (2008). Helminth 2-Cys peroxiredoxin drives Th2 responses through a mechanism involving alternatively activated macrophages. FASEB J..

[14] Reyes JL, Terrazas CA, Alonso-Trujillo J, van Rooijen N, Satoskar AR, Terrazas L (2010). Early removal of alternatively activated macrophages leads to *Taenia crassiceps* cysticercosis clearance *in vivo*. Int J Parasitol..

[15] Anthony RM, Urban JF, Alem F, Hamed HA, Rozo CT (2006). Memory T(H)2 cells induce alternatively activated macrophages to mediate protection against nematode parasites. Nat Med..

[16] Dzik JM, Golos B, Jagielska E, Zielinski Z, Walajtys-Rode E (2004). A non-classical type of alveolar macrophage response to *Trichinella spiralis* infection. Parasite Immunol..

[17] Donnelly S, O’Neill SM, Sekiya M, Mulcahy G, Dalton JP (2005). Thioredoxin peroxidase secreted by *Fasciola hepatica* induces the alternative activation of macrophages. Infect Immun..

[18] Xu J, Zhang H, Chen L, Zhang D, Ji M, Wu H, Wu G (2014). *Schistosoma japonicum* infection induces macrophage polarization. J Biomed Res..

[19] Zhu J, Xu Z, Chen X, Zhou S, Zhang W, Chi Y (2014). Parasitic antigens alter macrophage polarization during *Schistosoma japonicum* infection in mice. Parasites Vectors..

[20] Khelifi L, Soufli I, Labsi M, Touil-Boukoffa C (2017). Immune-protective effect of echinococcosis on colitis experimental model is dependent of down regulation of TNF-α and NO production. Acta Trop..

[21] Vendelova E, Hrckova G, Lutz MB, Brehm K, Nono JK (2016). *In vitro* culture of *Mesocestoides corti* metacestodes and isolation of immunomodulatory excretory-secretory products. Parasite Immunol..

[22] Hewitson JP, Grainger JR, Maizels RM (2009). Helminth immunoregulation: the role of parasite secreted proteins in modulating host immunity. Mol Biochem Parasitol..

[23] Hsu TL, Lin G, Koizumi A, Brehm K, Hada N, Chuang PK (2013). The surface carbohydrates of the *Echinococcus granulosus* larva interact selectively with the rodent Kupffer cell receptor. Mol Biochem Parasitol..

[24] Pan W, Xu HW, Hao WT, Sun FF, Qin YF, Hao SS (2018). The excretory-secretory products of *Echinococcus granulosus* protoscoleces stimulated IL-10 production in B cells *via* TLR-2 signaling. BMC Immunol..

[25] Ranasinghe SL, McManus DP (2018). *Echinococcus granulosus*: cure for cancer revisited. Front Med.

[26] Wang Y, Zhou H, Shen Y, Wang Y, Wu W, Liu H (2015). Impairment of dendritic cell function and induction of CD4^+^CD25^+^Foxp3^+^ T cells by excretory-secretory products: a potential mechanism of immune evasion adopted by *Echinococcus granulosus*. BMC Immunol..

[27] Kanan JH, Chain BM (2006). Modulation of dendritic cell differentiation and cytokine secretion by the hydatid cyst fluid of *Echinococcus granulosus*. Immunology..

[28] Wang Y, Wang Q, Lv S, Zhang S (2015). Different protein of *Echinococcus granulosus* stimulates dendritic induced immune response. Parasitology..

[29] Pan W, Hao WT, Shen YJ, Li XY, Wang YJ, Sun FF (2017). The excretory-secretory products of *Echinococcus granulosus* protoscoleces directly regulate the differentiation of B10, B17 and Th17 cells. Parasites Vectors..

[30] Amri M, Touil-Boukoffa C (2015). A protective effect of the laminated layer on *Echinococcus granulosus* survival dependent on upregulation of host arginase. Acta Trop..

[31] Li J, Zhang WB, Loukas A, Lin RY, Ito A, Zhang LH (2004). Functional expression and characterization of *Echinococcus granulosus* thioredoxin peroxidase suggests a role in protection against oxidative damage. Gene..

[32] Diaz A, Casaravilla C, Barrios AA, Ferreira AM (2016). Parasite molecules and host responses in cystic echinococcosis. Parasite Immunol..

[33] Cui SJ, Xu LL, Zhang T, Xu M, Yao J, Fang CY (2013). Proteomic characterization of larval and adult developmental stages in *Echinococcus granulosus* reveals novel insight into host–parasite interactions. J Proteomics..

[34] Virginio VG, Monteiro KM, Drumond F, de Carvalho MO, Vargas DM, Zaha A (2012). Excretory/secretory products from *in vitro*-cultured *Echinococcus granulosus* protoscoleces. Mol Biochem Parasitol..

[35] Zeghir-Bouteldja R, Polome A, Bousbata S, Touil-Boukoffa C (2017). Comparative proteome profiling of hydatid fluid from Algerian patients reveals cyst location-related variation in *Echinococcus granulosus*. Acta Trop..

[36] Wang H, Li J, Zhang C, Guo B, Wei Q, Li L (2018). *Echinococcus granulosus sensu stricto*: silencing of thioredoxin peroxidase impairs the differentiation of protoscoleces into metacestodes. Parasite..

[37] Hallowell RW, Collins SL, Craig JM, Zhang Y, Oh M, Illei PB (2017). mTORC2 signalling regulates M2 macrophage differentiation in response to helminth infection and adaptive thermogenesis. Nat Commun..

[38] Byles V, Covarrubias AJ, Ben-Sahra I, Lamming DW, Sabatini DM, Manning BD (2013). The TSC-mTOR pathway regulates macrophage polarization. Nat Commun..

[39] Zhang WB, Jones MK, Li J, McManus DP (2005). *Echinococcus granulosus*: pre-culture of protoscoleces *in vitro* significantly increases development and viability of secondary hydatid cysts in mice. Exp Parasitol..

[40] Wang H, Li J, Guo B, Zhao L, Zhang Z, McManus DP (2016). *In vitro* culture of *Echinococcus multilocularis* producing protoscoleces and mouse infection with the cultured vesicles. Parasites Vectors..

[41] Wang H, Li J, Pu H, Hasan B, Ma J, Jones MK (2014). *Echinococcus granulosus* infection reduces airway inflammation of mice likely through enhancing IL-10 and down-regulation of IL-5 and IL-17A. Parasites Vectors..

[42] Xu ZP, Chang H, Ni YY, Li C, Chen L, Hou M (2019). *Schistosoma japonicum* infection causes a reprogramming of glycolipid metabolism in the liver. Parasites Vectors..

[43] Noel W, Raes G, Hassanzadeh Ghassabeh G, De Baetselier P, Beschin A (2004). Alternatively activated macrophages during parasite infections. Trends Parasitol..

[44] Herbert DR, Holscher C, Mohrs M, Arendse B, Schwegmann A, Radwanska M (2004). Alternative macrophage activation is essential for survival during schistosomiasis and downmodulates T helper 1 responses and immunopathology. Immunity..

[45] Reyes JL, Terrazas LI (2007). The divergent roles of alternatively activated macrophages in helminthic infections. Parasite Immunol..

[46] Thompson RC, Jenkins DJ (2014). *Echinococcus* as a model system: biology and epidemiology. Int J Parasitol..

[47] Nakao M, Lavikainen A, Yanagida T, Ito A (2013). Phylogenetic systematics of the genus *Echinococcus* (Cestoda: Taeniidae). Int J Parasitol..

[48] Tamarozzi F, Mariconti M, Neumayr A, Brunetti E (2016). The intermediate host immune response in cystic echinococcosis. Parasite Immunol..

[49] Diaz A, Fernandez C, Pittini A, Seoane PI, Allen JE, Casaravilla C (2015). The laminated layer: recent advances and insights into *Echinococcus* biology and evolution. Exp Parasitol..

[50] Soufli I, Toumi R, Rafa H, Amri M, Labsi M, Khelifi L (2015). Crude extract of hydatid laminated layer from *Echinococcus granulosus* cyst attenuates mucosal intestinal damage and inflammatory responses in dextran sulfate sodium induced colitis in mice. J Inflamm.

[51] Seoane PI, Ruckerl D, Casaravilla C, Barrios AA, Pittini A, MacDonald AS (2016). Particles from the *Echinococcus granulosus* laminated layer inhibit IL-4 and growth factor-driven Akt phosphorylation and proliferative responses in macrophages. Sci Rep..

[52] Soroosh P, Doherty TA, Duan W, Mehta AK, Choi H, Adams YF (2013). Lung-resident tissue macrophages generate Foxp3^+^ regulatory T cells and promote airway tolerance. J Exp Med..

[53] Kearley J, Barker JE, Robinson DS, Lloyd CM (2005). Resolution of airway inflammation and hyperreactivity after *in vivo* transfer of CD4^+^CD25^+^ regulatory T cells is interleukin 10 dependent. J Exp Med..

[54] Fukao T, Tanabe M, Terauchi Y, Ota T, Matsuda S, Asano T (2002). PI3K-mediated negative feedback regulation of IL-12 production in DCs. Nat Immunol..

[55] Ohtani M, Hoshii T, Fujii H, Koyasu S, Hirao A, Matsuda S (2012). Cutting edge: mTORC1 in intestinal CD11c^+^ CD11b^+^ dendritic cells regulates intestinal homeostasis by promoting IL-10 production. J Immunol..

[56] Weichhart T, Costantino G, Poglitsch M, Rosner M, Zeyda M, Stuhlmeier KM (2008). The TSC-mTOR signaling pathway regulates the innate inflammatory response. Immunity..

[57] Yang CS, Song CH, Lee JS, Jung SB, Oh JH, Park J (2006). Intracellular network of phosphatidylinositol 3-kinase, mammalian target of the rapamycin/70 kDa ribosomal S6 kinase 1, and mitogen-activated protein kinases pathways for regulating mycobacteria-induced IL-23 expression in human macrophages. Cell Microbiol..

[58] Covarrubias AJ, Aksoylar HI, Horng T (2015). Control of macrophage metabolism and activation by mTOR and Akt signaling. Semin Immunol..

