# Supplementary material for: Thioredoxin peroxidase secreted by Echinococcus granulosus (sensu stricto) promotes the alternative activation of macrophages via PI3K/AKT/mTOR pathway
Source: Parasit Vectors. 2019 Nov 14;12:542. doi: 10.1186/s13071-019-3786-z (PMC6857240; doi:10.1186/s13071-019-3786-z)
Supplement: Supplementary file 1 — Additional file 1: Table S1. Antibodies utilized in the flow cytometry analysis. [file 13071_2019_3786_MOESM1_ESM.doc]

Additional file 1: Table S1. Antibodies utilized in the flow cytometry analysis

| **Antibody** | **Clone** | **Fluorochrome** | **From** |
| --- | --- | --- | --- |
| CD45 | 30-F11 | PerCP/Cy5.5 | Biolegend |
| CD3 | 17A2 | FITC | Biolegend |
| NK1.1 | PK136 | FITC | Biolegend |
| CD19 | 6D5 | FITC | Biolegend |
| CD11b | M1/70 | Brilliant Violet 650 | Biolegend |
| F4/80 | BM8 | PE | Biolegend |
| MHCII | M5/114.15.2 | Brilliant Violet 510 | Biolegend |
| CD80 | 16-10A1 | PE-Cyanine 7 | Biolegend |
| CD86 | GL-1 | APC | Biolegend |
| CD206 | 15-2 | APC | Biolegend |
| iNOS | CXNFT | PE-Cyanine 7 | eBioscience |
