# Supplementary figures and images for: Thioredoxin peroxidase secreted by Echinococcus granulosus (sensu stricto) promotes the alternative activation of macrophages via PI3K/AKT/mTOR pathway
Source: Parasit Vectors. 2019 Nov 14;12:542. doi: 10.1186/s13071-019-3786-z (PMC6857240; doi:10.1186/s13071-019-3786-z)

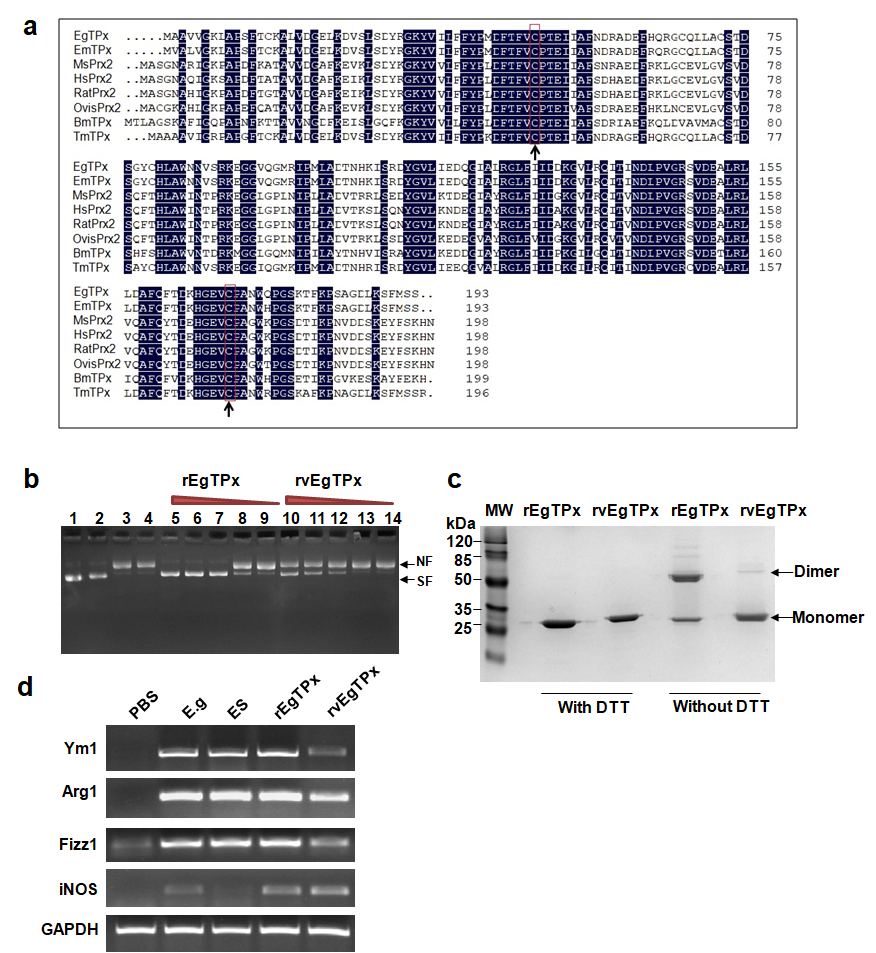

Supplement: Supplementary file 2 — Additional file 2: Figure S1. Functional expression and characterization of recombinant EgTPx. a Multiple sequence alignment of EgTPx (AAL84833), E. multilocularis TPx (EmTPx; BAC11863), mouse Prx2 (MsPrx2; Q61171), human Prx2 (HsPrx2; P32119), Rattus Prx2 (RatPrx2; NP_058865), Ovis aries Prx2 (OvisPrx2; NP_001159672), Brugia malayi TPx (BmTPx; Q17172) and Taenia multiceps TPx (TmTPx; ADW77118). The arrows indicate the redox-active Cys48 and Cys169 residues that were replaced by Gly to generate EgTPx variants (rvEgTPx). b Protection of plasmid DNA from oxidative damage by rEgTPx in MCO system. M: DNA marker; Lane 1: DNA alone; Lane 2: DNA in water with incubation; Lane 3: DNA in MCO system with incubation; Lane 4: DNA in MCO system incubated with BSA; Lanes 5–9 and 10–14: DNA in MCO system incubated with 400, 200, 100, 25 and 12.5 μg/ml of rEgTPx and rvEgTPx, respectively; NF, nicked form of the plasmid; SF, supercoiled form of the plasmid. c rEgTPx and rvEgTPx were electrophoresed under reducing (with DTT) and nonreducing conditions (without DTT). The monomeric and dimeric forms of the enzymes are indicated by arrows. d RT-PCR was used to assess the expression of Ym1, Arg1, Fizz1 and iNOS in PMs isolated from E. granulosus (s.s.)-infected and parasite antigen-treated mice. [file 13071_2019_3786_MOESM2_ESM.tif]

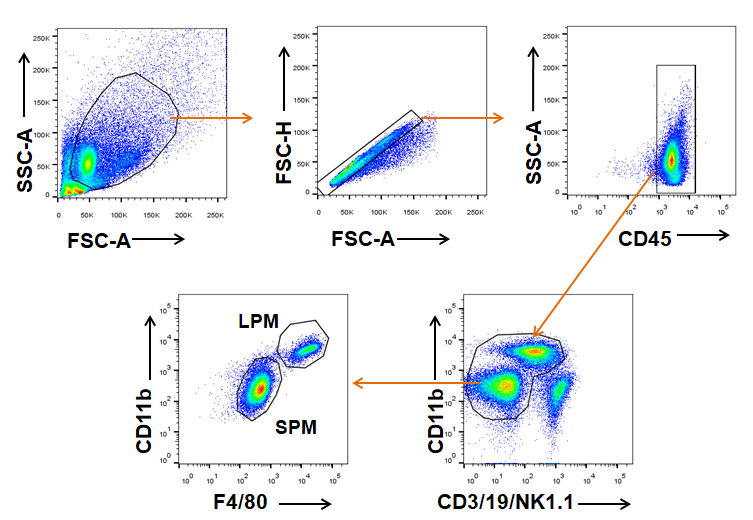

Supplement: Supplementary file 3 — Additional file 3: Figure S2. Gating strategy used for the identification of large and small peritoneal macrophages (LPMs and SPM). LPMs: CD45+CD3−CD19−NK1.1− and CD11bhighF4/80high. SPMs: CD45+CD3−CD19−NK1.1− and CD11blowF4/80low. [file 13071_2019_3786_MOESM3_ESM.tif]

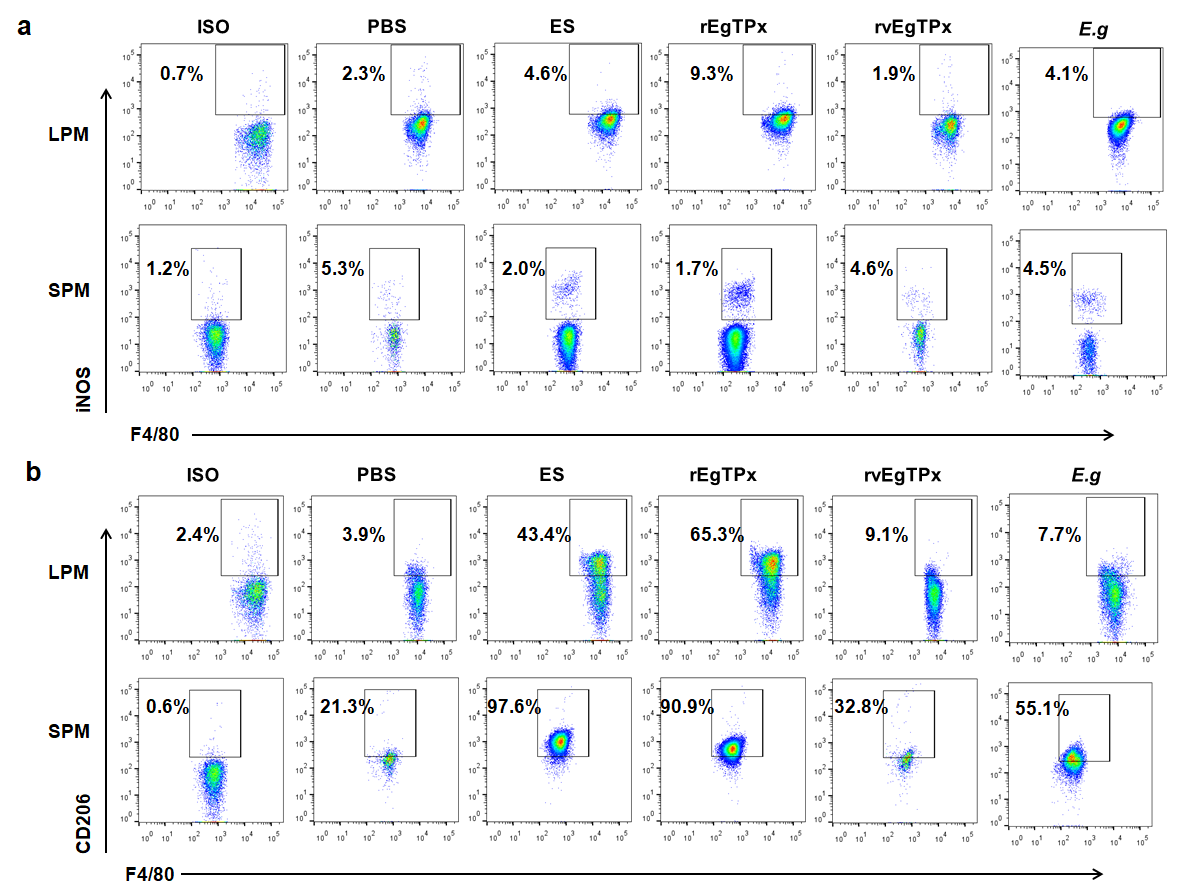

Supplement: Supplementary file 4 — Additional file 4: Figure S3. Representative FACS plots gated on PM subsets from E. granulosus (s.s.)-infected and parasite antigen-treated mice. a, b Intracellular staining of iNOS+ and CD206+ in LPMs and SPMs, respectively. [file 13071_2019_3786_MOESM4_ESM.tif]

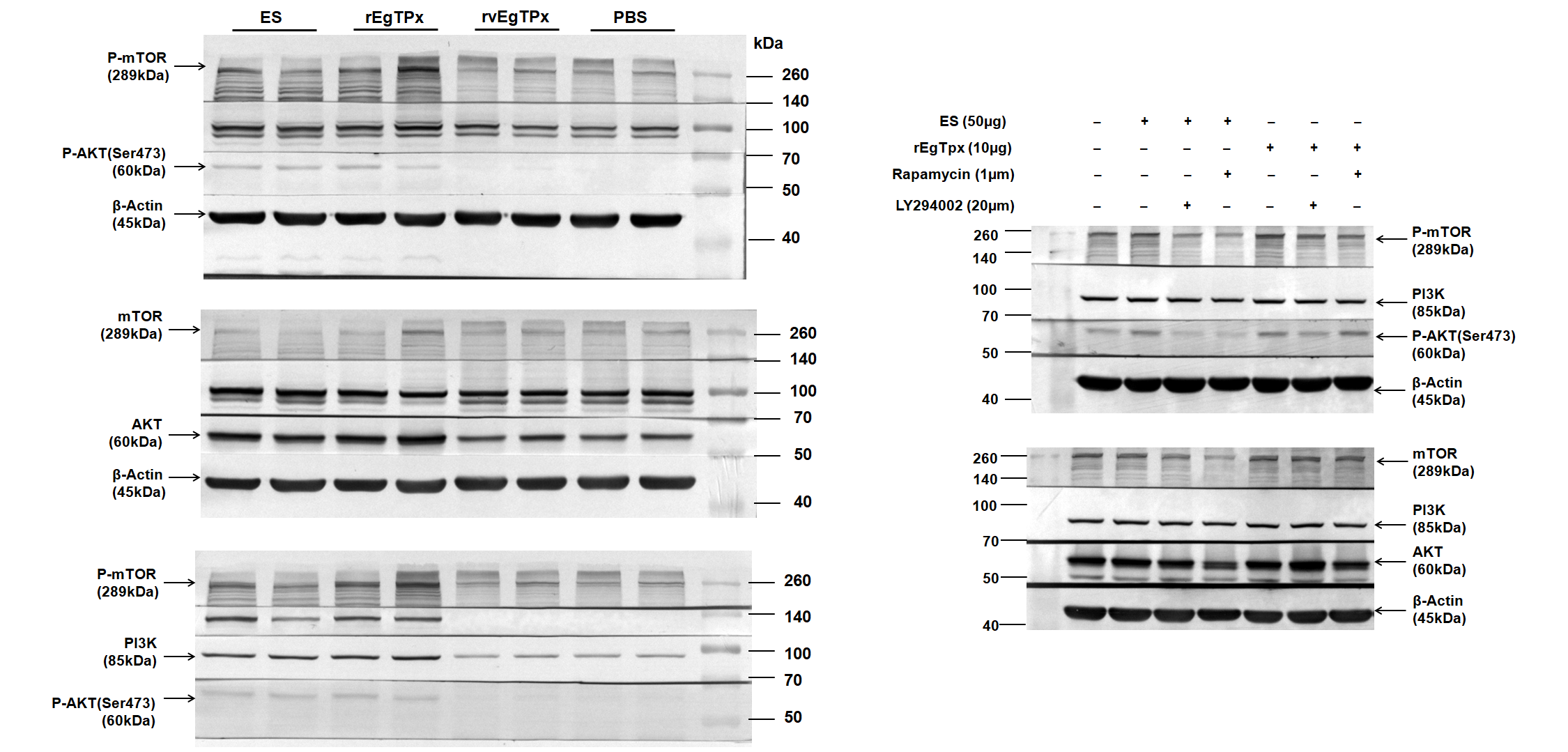

Supplement: Supplementary file 5 — Additional file 5: Figure S4. Uncropped Western blots corresponding to the results shown in Fig. 5. [file 13071_2019_3786_MOESM5_ESM.tif]
